# High Touch and High Tech (HT2) Proposal: Transforming Patient Engagement Throughout the Continuum of Care by Engaging Patients with Portal Technology at the Bedside

**DOI:** 10.2196/resprot.6355

**Published:** 2016-11-29

**Authors:** Ann Scheck McAlearney, Cynthia J Sieck, Jennifer L Hefner, Alison M Aldrich, Daniel M Walker, Milisa K Rizer, Susan D Moffatt-Bruce, Timothy R Huerta

**Affiliations:** ^1^ Research Division Department of Family Medicine The Ohio State University Columbus, OH United States; ^2^ Information Technology Wexner Medical Center Ohio State University Columbus, OH United States; ^3^ Clinical Division Department of Family Medicine The Ohio State University Columbus, OH United States; ^4^ Department of Biomedical Informatics College of Medicine The Ohio State University Columbus, OH United States; ^5^ Quality & Patient Safety Wexner Medical Center The Ohio State University Columbus, OH United States; ^6^ Department of Surgery College of Medicine The Ohio State University Columbus, OH United States

**Keywords:** patient portals, personal health records, patient participation, patient engagement, patient access to records, patient satisfaction, mobile computing, care transitions, chronic disease, self-efficacy, hospitalization, ambulatory care

## Abstract

**Background:**

For patients with complex care needs, engagement in disease management activities is critical. Chronic illnesses touch almost every person in the United States. The costs are real, personal, and pervasive. In response, patients often seek tools to help them manage their health. Patient portals, personal health records tethered to an electronic health record, show promise as tools that patients value and that can improve health. Although patient portals currently focus on the outpatient experience, the Ohio State University Wexner Medical Center (OSUWMC) has deployed a portal designed specifically for the inpatient experience that is connected to the ambulatory patient portal available after discharge. While this inpatient technology is in active use at only one other hospital in the United States, health care facilities are currently investing in infrastructure necessary to support large-scale deployment. Times of acute crisis such as hospitalization may increase a patient’s focus on his/her health. During this time, patients may be more engaged with their care and especially interested in using tools to manage their health after discharge. Evidence shows that enhanced patient self-management can lead to better control of chronic illness. Patient portals may serve as a mechanism to facilitate increased engagement.

**Objective:**

The specific aims of our study are (1) to investigate the independent effects of providing both High Tech and High Touch interventions on patient-reported outcomes at discharge, including patients’ self-efficacy for managing chronic conditions and satisfaction with care; and (2) to conduct a mixed-methods analysis to determine how providing patients with access to MyChart Bedside (MCB, High Tech) and training/education on patient portals, and MyChart Ambulatory (MCA, High Touch) will influence engagement with the patient portal and relate to longer-term outcomes.

**Methods:**

Our proposed 4-year study uses a mixed-methods research (MMR) approach to evaluate a randomized controlled trial studying the effectiveness of a High Tech intervention (MCB, the inpatient portal), and an accompanying High Touch intervention (training patients to use the portal to manage their care and conditions) in a sample of hospitalized patients with two or more chronic conditions. This study measures how access to a patient portal tailored to the inpatient stay can improve patient experience and increase patient engagement by (1) improving patients’ perceptions of the process of care while in the hospital; (2) increasing patients’ self-efficacy for managing chronic conditions; and (3) facilitating continued use of a patient portal for care management after discharge. In addition, we aim to enhance patients’ use of the portal available to outpatients (MCA) once they are discharged.

**Results:**

This study has been funded by the Agency for Healthcare Research and Quality (AHRQ). Research is ongoing and expected to conclude in August 2019.

**Conclusions:**

Providing patients real-time access to health information can be a positive force for change in the way care is provided. Meaningful use policies require minimum demonstrated use of patient portal technology, most often in the ambulatory setting. However, as the technology matures to bridge the care transition, there is a greater need to understand how patient portals transform care delivery. By working in concert with patients to address and extend current technologies, our study aims to advance efforts to increase patients’ engagement in their care and develop a template for how other hospitals might integrate similar technologies.

## Introduction

### Background

#### The Burden of Chronic Illness and Multimorbidity

Chronic illnesses, including cardiovascular disease, arthritis, diabetes, asthma, cancer or chronic obstructive pulmonary disease (COPD), affect more than 145 million Americans, representing a large increase over projections made only 10 years ago [1-3]. The individual burden of chronic illness is magnified by the high rate of two or more co-occurring chronic illnesses, termed multimorbidity [1]. This population is at particularly high risk for a variety of adverse health outcomes including poor functional status, unnecessary hospitalizations, and adverse drug events [4-6]. Chronic illnesses account for 7 out of 10 deaths each year. In addition, chronic illness has resulted in huge costs to medical systems and society, accounting for 84% of the total US health care spending [1], or almost 15% of the gross domestic product (GDP). For these reasons the Agency for Health Care Research and Quality (AHRQ) has designated people with multimorbidity as a priority population [7].

#### Patient Self-Management Using Health Information Technologies

Individuals with chronic illness generally receive care in ambulatory settings and often look to primary care providers to direct their care. However, the rise in chronic illness is shifting the role of the physician to that of facilitator in the patient’s self-management process. The increase in availability of personal disease monitoring tools such as hand-held, self-monitoring blood glucose systems [8], and a focus on patients as consumers of health care is moving expectations around management of chronic conditions away from laboratories and physicians to the patients themselves. Evidence shows that enhanced patient self-management can lead to better control of chronic illness [9-11], and health information technology (HIT) is a potentially important mechanism to facilitate patient self-management [12-15]. Large studies in outpatient settings have found that providing patients with access to their medical record, physician progress notes, personalized health information, and reminders (ie, patient-centered functionalities) leads to increases in adherence to guidelines, health status, and patient satisfaction [16,17].

#### Tools for Managing Health

Patient portals are a class of electronic personal health records (PHRs)—tools that patients can use to track and manage their health. The PHR is “an electronic record of an individual’s health information by which the individual controls access to the information and may have the ability to manage, track, and participate in his or her own health care” [18]. PHRs can be stand-alone or tethered. Stand-alone products allow the patient full control over what data are entered and accessed and are independent of the provider, allowing the patient to input their own data from any provider regardless of the electronic health record (EHR) system (if any) the provider uses, and to carry their data with them across providers. Tethered PHRs, also called patient portals, are offered through a health care provider and are connected to the patient’s EHR with that provider. These patient portals provide access to information in the patient’s EHR, controlled by the provider, as well as other functions such as viewing and scheduling appointments and secure communication with the provider [19]. Both types of PHRs show promise for assisting patients in self-management of chronic conditions by allowing patients to input and track health information, facilitating communication between patients and providers, and providing access to consumer-friendly information about diseases [20-24].

Research has shown that patients with special health care needs, such as those with multiple chronic conditions, have the greatest interest in patient portals [25-27]. The first large-scale study of the use of patient portals within a large health system found that having more chronic conditions predicted both adoption and intensity of patient portal use [28]. However, there is still much to understand about why, how, and which patients use portals, and how the health care system can best support them. Currently, little is known about what motivates patients to adopt and continue to use portals, and what functionalities patients consider important for self-management of their conditions [13-15].

#### Patient Engagement Through Health Information Technologies

Patient engagement, defined by the Institute for Healthcare Improvement as “actions that people take for their health and to benefit from care” [29], is critical to the management of chronic diseases. A 2013 editorial in Health Affairs referred to patient engagement as the “next blockbuster drug of the century” [29]. Patient portals are positioned as a central component of patient engagement through the potential to change the physician-patient relationship and enable chronic disease self-management [30-33]. Studies of outpatient portals suggest that patients want accurate and timely information provided across the continuum of care that they can apply to their care and communicate with providers in a secure and trusted manner [14,34].

Despite the focus on patient engagement [29,35], research on patient engagement in the inpatient setting is in its infancy. A recent systematic review found only four studies testing the provision of patient-specific information in the inpatient setting [36]. The focus of these studies included providing access to the patient record and information on the care team through a mobile phone app [37], a tablet computer app to view care team profiles and hospital medication records, a tablet app with the plan of care, diet and safety information [38], and large in-room information displays in an emergency department [39]. While these small-scale qualitative case studies reported positive findings, including patient reports of enhanced engagement in the care process and satisfaction with care, none included patient-centered functionality such as the ability to send messages to the care team, allowing patients to input information or record notes—elements that have been demonstrated to further enhance patients’ engagement [17,40,41].

#### Increasing Motivation for Patient Engagement

Information and technology are insufficient to fully engage patients in their care; patients also need motivation to engage [42-46]. A common element of health behavior change theories is the need for a trigger to action [47,48]. This is supported in studies of individual behavior change across a variety of health behaviors [49-54]. For patients with multiple chronic conditions, hospitalization is often due to exacerbations of one or more condition. We assert that hospitalization can serve as the necessary trigger that engages these patients in managing their care [47,48]. In other areas, times of acute crisis have been linked to a greater perception of risk and increased focus on health behaviors [49-52]. Therefore, hospitalization may create a window of higher engagement in which to initiate behavior change and foster interest in tools for managing health.

#### Addressing Barriers to Patient Portal Use

Most patient portal implementations have assumed that internal documentation is sufficient, and that the application is sufficiently intuitive for use with only a supplemental list of “frequently asked questions (FAQs)”; however, documentation and FAQs alone are not always sufficient (see Pilot Study section). In particular, individuals with low health literacy and low technological literacy may experience greater difficulty navigating these tools and may simply give up. The Institute of Medicine defines health literacy as “the degree to which individuals have the capacity to obtain, process, and understand basic health information and services needed to make appropriate health decisions” [55]. Electronic health (eHealth) literacy, then, is “the ability to seek, find, understand, and appraise health information from electronic sources and apply the knowledge gained to addressing or solving a health problem” [56]. Technological literacy, including knowing how to operate computers and mobile devices, and how to make use of the information provided, are essential components of eHealth literacy.

The multimorbid tend to be older and more socioeconomically disadvantaged than the population at large [57], so people with multiple chronic illnesses may be especially challenged in developing eHealth literacy skills. However, people of all ages and status struggle with health literacy. Poor health literacy is a better predictor of poor health status than income, employment status, or education [55]. Interventions thus need to provide training specifically designed to overcome these challenges. Further, the introduction of new technology into the care process, such as tablet computers, may create barriers to participation due to the competing demands on a patient’s time inherent in hospitalization. If these tools are to accomplish their intended aims of empowering patients through greater engagement, it is critical to understand the training and support materials necessary to meet diverse patient needs.

### High Tech/High Touch: Our Proposed HT2 Project

We propose the first large-scale randomized controlled trial (RCT) of the impact and use of an inpatient tablet-based patient portal including a mixed methods analysis. This study examines changes in patient self-efficacy managing chronic conditions and subsequent ambulatory patient portal usage as well as associated experiences and outcomes. We will explore outcome differences across two concurrent dimensions: the provision of an inpatient patient portal for multimorbid patients and a training intervention focused on using this HIT to improve self-management. The resultant 2x2 experimental design offers the ability to measure how comfort with technology moderates the use of technology to manage chronic conditions by comparing results from differently structured Tech and Touch interventions. By addressing literacy through high and low touch intervention approaches, we will explore the value that different engagement approaches provide. Follow-up with patients post discharge will enable us to study how the inpatient experience influences ongoing usage of similar linked tools in the ambulatory setting.

Our Tech Intervention utilizes the MyChart Bedside (MCB) and MyChart Ambulatory (MCA) patient portals to assist patients in managing their chronic conditions. MCB is an inpatient portal patients can use to access their data while at an Epic-equipped hospital that has deployed the technology. MCB was developed by Epic to provide patients, and their families and caregivers, access to information customized to the inpatient setting. It includes an expected care plan for the day, health education materials, secure messaging with the care team, a place to take notes, and access to educational videos.

MCA is a Web-based ambulatory patient portal providing access to similar data, but is focused on outpatient care functions. MCA includes access to a health summary, medication listing, immunizations, patient health data entry (eg, submission of daily glucose levels), appointment tracking, secure messaging, management of preventive care, and information associated with financial management of the patient account. MCA is available from any computer with an Internet connection and a browser, and from a mobile app for iOS (Apple) and Android devices.

MCB and MCA access different elements of the medical record, and as such, are independent apps used at different times across the continuum of care. However, because the same EHR underlies both systems, there is significant overlap in the information available in each app. Patients using MCB are prompted to create an MCA account at discharge if they do not already have one. Information from a hospital stay, including all lab and test results, is available in MCA within 3 days of posting to the EHR so that patients with an MCA account will have access to their MCB information after discharge. Together, these apps have the potential to provide patients with access to the right information at the right time across the continuum of care.

Our Touch intervention provides training to patients in both the technical and application aspects of using a patient portal to manage their chronic conditions. The High Touch intervention will involve in-person guidance from a technology navigator (TN) to educate patients about the use of MCA (Low Tech) or both MCA and MCB (High Tech). The subject of this discussion will follow a predetermined script explaining various tasks, engaging patients with the portal application by allowing them to complete tasks in the presence of a TN, and addressing any issues, concerns, or questions that arise during the intervention. In contrast, patients assigned to the Low Touch intervention are presented an initial video after consent that explains the use of MCA (Low Tech) or both MCA and MCB (High Tech). In both cases, the TN will provide patients with paper-based signup and support materials on the use of MCA prior to discharge. In the High Touch case, the emphasis is on providing one-on-one training in the use of MCA (Low Tech) or both MCA and MCB (High Tech) at admission with a follow-up at discharge. While we have a preliminary design for this intervention, we expect that the tools will experience at least one iteration of improvement, and therefore our proposed project includes an initial usability study to identify timely information for the intervention.

### Pilot Studies

In preparation for this project, the research team conducted extensive pilot studies, engaged in tool development, and promoted practice change and quality improvement to support the proposed study. Selected pilot projects are described in the following sections.

#### MyChart Bedside Use

We initiated a pilot study in 4 units within Ohio State University Wexner Medical Center (OSUWMC) that tested the specifications for implementation of MCB. This preliminary study served to formatively guide our High Tech/High Touch (HT2) proposal. For instance, the pilot identified the need for a High Touch intervention, the need to use TNs (rather than existing staff) to provision tablets, and the need for easily accessible support materials. Videos created for this pilot serve as the initial basis for our Low Touch intervention, and we will make modifications as needed for our study purposes.

#### MyChart Ambulatory Use

In preparation for our proposed HT2 study, we secured ongoing report data on the use of MCA. From these data we know that the mean age of MCA users is 47.6 years (SD=16); and that most are white, have managed care insurance, and have used MCA for nearly 2 years. It was found that 44.82% (31,439/70,152) of all patients created a MCA account; however, only 16.00% (5029/31,439) had logged on and used their account during the pilot study time frame. This pilot served to reinforce our need to focus on the use of functions within MCA and MCB, rather than the standard model promoting account sign up and leading to only intermittent use.

#### Usability Studies in Epic

Dr Huerta has been assessing usability of functions within Epic. Working with students to engage health practitioners, preliminary work was conducted to explore the usability of the system. Pilot research results have recently been made available to the health system.

### Rationale for Study Design

The current state of the literature regarding patient portals is primarily based on observational or small-scale qualitative studies [13]. We propose to use a randomized controlled design to isolate the impact associated with the adoption of an inpatient patient portal (MCB), and a concomitant intervention addressing comfort with technology and health literacy. The study is of sufficient size to identify subgroup dynamics, as well as explore whether ambulatory patient portal usage (MCA) is influenced by the availability and usage of the inpatient portal. Our study will also examine how use patterns may impact health outcomes (eg, readmission rates), patient-reported outcomes (eg, perceived self-efficacy in chronic disease management), and patients’ experiences (eg, satisfaction with care).

#### Focus on Multimorbidity Patients

By focusing specifically on engaging patients with multimorbidity in the use of patient portals across the continuum of care, HT2 addresses one of the main goals set forth in the 2010 Department of Health and Human Services report, Multiple Chronic Conditions: A Strategic Framework [4], to implement and effectively use health care technology. As multimorbidity research is not disease-specific, our study focuses on self-efficacy and satisfaction as the outcomes of interest because they have been associated in previous research with positive health outcomes [7] and are considered generic outcome measures that are responsive to change over time [6]. Current studies of patient portal use focus mainly on metrics such as number of users and email response times, but these do not necessarily reflect use in a way that impacts an individual’s health. We plan to study the potential for how introducing patient portals during an inpatient stay can influence patient-centered care and outcomes including patients’ perceived self-efficacy for managing chronic conditions across the continuum of care. Further, we expect to be able to examine both readmission rates and rates of ambulatory patient portal utilization (MCA) after discharge in this priority patient population.

#### Potential to Increase Patient Engagement in Disease Self-Management and the Process of Care

Our study also highlights the importance of patients’ engagement in their care. Patient portals offer several tools to help patients become and remain engaged. In the inpatient setting, when patients may be particularly ready to learn about managing their health, patients can learn how and why it is important to track biometric measures such as weight or blood glucose levels, receive feedback from and ask questions of their providers specific to the management of their conditions, and access disease-specific educational materials. They can also send secure messages to their hospital and family physicians. This communication opportunity has the potential to change patient engagement with their nursing team as well. In our MCB pilot study (discussed below), nursing staff considered it important to disseminate educational material throughout the hospital stay rather than simply at discharge, because this allows education to happen when the patient is ready to engage. These same materials can then be made available in MCA when the patient leaves the hospital. With this study, we seek to clarify and measure how engagement in the inpatient setting (use of MCB) facilitates continued engagement once the patient leaves the hospital (ongoing use of MCA). At issue is whether use during an inpatient stay reinforces ambulatory use.

#### Study Results Are Likely to Improve Health Care and Outcomes

Our study aims to improve health care and outcomes through implementation of enhanced patient-centered HIT. The integration of patient portals within EHR systems has the potential to improve the patient experience and the quality of patient care [28,58-60]. However, use of patient portals beyond creating an initial account remains limited, and is based on the technology's relevance to the patient and the ability of the technology to enhance the physician-patient relationship [34,61,62]. Our study focuses on increasing relevance and improving communication to promote patient portal use in order to improve care, outcomes, and experience for patients with multiple chronic conditions. We specifically explore the impact of education strategies as a means to compensate for differences in technology and health literacy. In doing so, we intend to explore the use of both technology and literacy training to increase self-efficacy in the use of these tools. Evidence shows that enhanced patient self-efficacy for management activities can lead to better control of chronic illness [9-11].

#### Widespread Reach of Epic

The patient portals to be studied at OSUWMC are available through the Epic EHR system in use at Ohio State. Epic currently touches over 50% of all Americans as they receive health care and is available to 50 million patients in the United States [20,63]. Both MCA and MCB patient portals are integrated into the Epic EHR at OSUWMC.

## Methods

### Approach Overview

Our study uses a mixed methods approach to examine an inpatient patient portal called MyChart Bedside (MCB) combined with patient-specific training. We use a 2x2 experimental design within an RCT to study both use and impacts of MCB. We compare levels of Tech and Touch both independently and together to improve understanding of both short- and long-term effects at the critical time for behavioral change – hospitalization. The large sample size (N=6000) provides an opportunity to effectively engage in subgroup analysis.

### Conceptual Framework and Study Hypotheses: The Health Belief Model

The Health Belief Model [47] provides a context for understanding why the use of a patient portal during an inpatient stay may increase engagement in managing chronic conditions in the future. This model suggests that the likelihood of a person engaging in a health-related behavior, such as managing a chronic illness, is based on (1) perceptions of factors such as risk, seriousness, and their own ability to make that change; and (2) aspects of the environment that might trigger taking action. First, the patient’s assessment and understanding of the seriousness of their condition and consequences of not addressing it, combined with how susceptible they believe they are to the consequences of their condition, influence how much of a threat the person perceives from not taking action. Perceived threat, along with the person’s assessment of the benefits and barriers to taking action, and their confidence that they can take that action (called self-efficacy) are then expected to influence how likely a person is to take a health-related action. In the person’s environment, cues to action can provide additional motivation to take action. We posit that hospital admission can serve as one of these cues to action.

With diabetes, for example, a health-promoting action a patient can take is monitoring his blood sugar level. According to the Health Belief Model, the likelihood of taking this action would be influenced by several factors including (1) how serious the patient perceives the consequences of not monitoring their blood sugar (eg, hospitalization); (2) their perceived risk of experiencing those consequences; (3) what benefits they might experience from monitoring (eg, more even blood sugar control); (4) barriers to monitoring (eg, painful finger sticks); (5) how confident they feel in monitoring their blood sugar; and (6) reminders in their environment of the need to monitor. Training a patient to monitor their blood sugar after discharge and communicate that information to his/her physician can reduce the likelihood of readmission for uncontrolled diabetes.

While the hospital experience is often about moving a patient out of crisis, it also represents an opportunity to influence the patient’s assessment of the benefits and barriers to taking action (seriousness and risk), and their confidence that they would be able to achieve the necessary behavioral change required to achieve the desired consequence (self-efficacy). Patient portals in this setting offer tools to increase self-efficacy during a particularly receptive time that can be continued in the outpatient environment. Our intervention provides not only the tools for continued management, but patient-centered training in their use as well.

### Study Hypotheses

**Figure 1:**
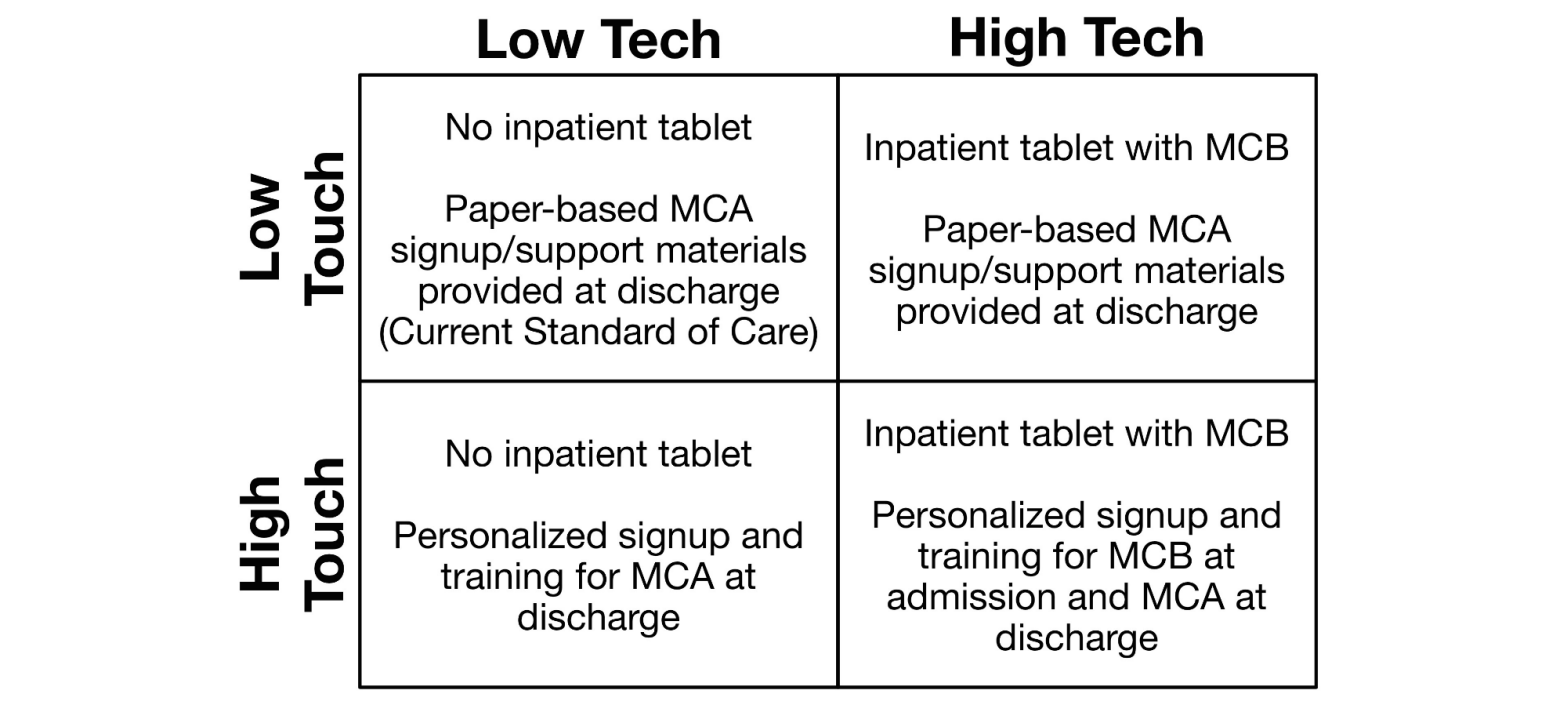
Study design. MCA: MyChart Ambulatory; MCB: MyChart Bedside.

We expect that participants in each of the experimental groups (see Figure 1) will have a fundamentally different experience as a result of the intervention arms. As such, we have generated 7 (H1 to H7) hypotheses (Textbox 1). Within these general hypotheses, we will also explore effects among different subgroups including variations based on health literacy, computer self-efficacy, health conditions, and socioeconomic status.

**Study hypotheses.**

H1: Patients with access o MyChart Bedside (MCB) (High Tech) will report higher satisfaction with care experience, greater changes in self-efficacy, and fewer readmissions than those who did not have MCB access.

H2: Patients who receive in-person training interventions (High Touch) will report higher satisfaction with care and greater changes in comfort with technology than those who were not provided with in-person training.

H3: An interaction effect will exist between High Tech and High Touch, such that the provision of both will result in better experiences than the provision of only one intervention component.

H4: Use of patient education materials within MCB will be linked to greater perceived self-efficacy for patients’ management of chronic disease.

H5: Across all patients, patients with increased use of technology (High Tech) will experience lower readmission rates.

H6: Patients with access to MCB (High Tech) will be more likely to use MyChart Ambulatory (MCA) more often and with greater intensity and have better experiences in primary care (ie, higher patient satisfaction), controlling for preadmission MCA use (available only to patients who use Ohio State University Wexner Medical Center [OSUWMC] providers as outpatients).

H7: Patients with training in both MCA and MCB (High Tech) will use MCA more often and with greater intensity, and have better experiences in primary care than those who were provided access to both but no training.

**Figure 2:**
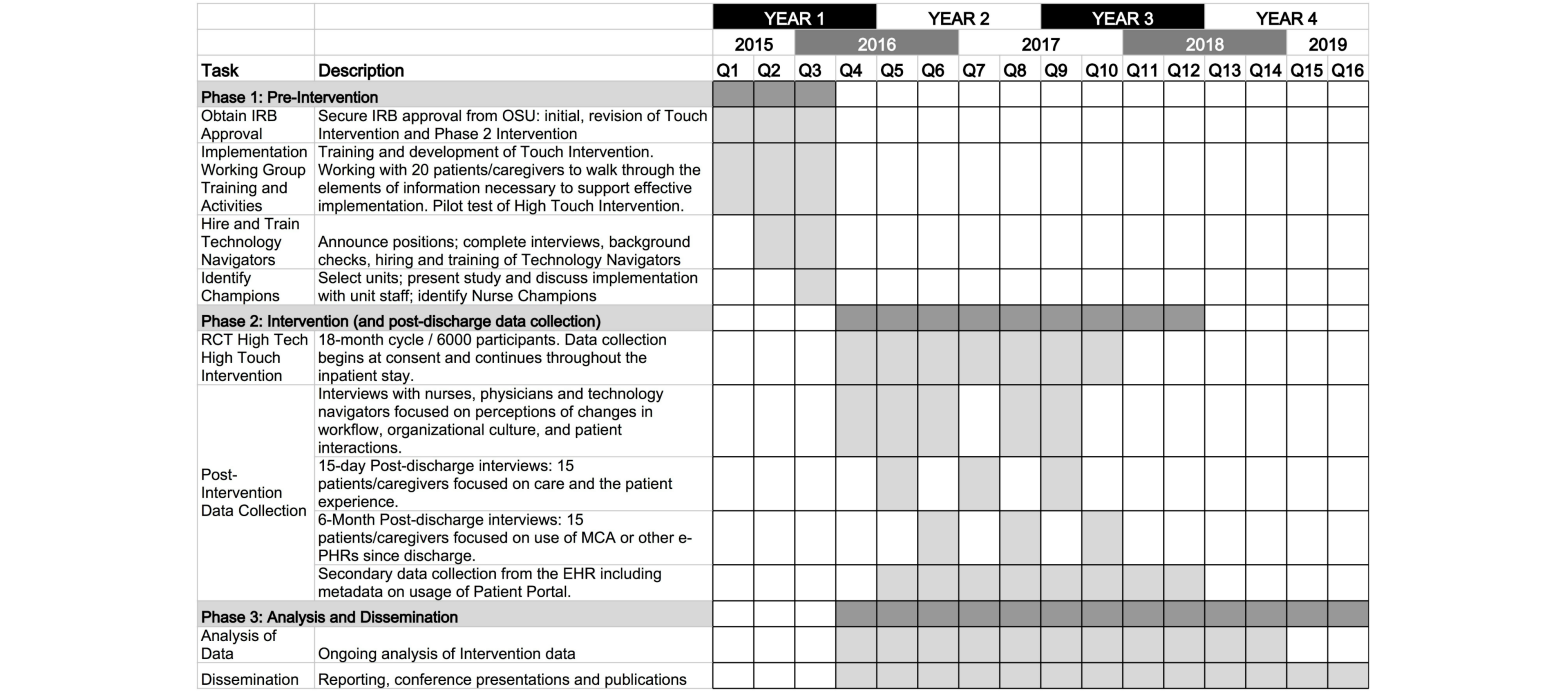
Project timeline. IRB: institutional review board; OSU: Ohio State University; RCT: randomized controlled trial; MCA: MyChart Ambulatory; PHR: personal health record; EHR: electronic health record.

**Inclusion criteria.**

1. Patient admitted in the last 24 hours.

2. Expected discharge is more than 3 days away (over 72 hours); the average length stay at Ohio State University Wexner Medical Center (OSUWMC) is currently 5.4 days.

3. Two or more chronic conditions.

4. Patient is available in room.

5. Patient is capable of providing informed consent.

### Study Design

We have delineated 4 study arms that vary based on the level of the Tech and Touch interventions planned (Figure 1). We have selected an experimental design that will allow us to explore the impact of providing supportive patient portal training as well as technology, but separate and apart from one another. The Touch interventions will be designed to provide approximately equivalent in-person time with a TN in order to control for bias. The Low Touch/Low Tech group was designed to allow for interaction with the TN but in a manner that should not affect the assessment model. Only patients assigned to the research study and selected for the High Tech intervention will be able to create accounts using MCB while they are hospitalized.

#### Study Population and Setting

The study randomizes individual patients within 1 of 6 general medicine units from OSUWMC into 1 of 4 study arms (Figure 1). While the unit of analysis and intervention is the patient, absent unit level controls, the study would suffer from endogeneity issues related to unit workflow dynamics that are changed by the presence of the tools, and as such the use of a unit-level control is justified. We will identify 6 matched pairs of general medicine units (12 total) on the basis of Case Mix Index, average length of stay, number of beds, nurse staffing levels and “nurse to bed staffing ratio,” and select units for the intervention in each pair. Matched controls offer the ability to calculate a de-identified reference cohort of patients against which readmission and MCA uptake can be measured. In addition, we will conduct interviews with a purposive sample of health care professionals across the system to examine how the roll out of MCB impacts patient discussions in both the inpatient and ambulatory care settings; these interviews will include topics such as expectations of MCA usage, and perceptions of the technology and touch interventions.

#### Sample Size and Power Calculations

Sample size and power calculations were made using current patient satisfaction scores as a baseline for each measure using G*Power 3.1 software. We expect this design to be sensitive enough to identify changes of between 2.2% and 3.8% across the panel of patient satisfaction scores in the Hospital Consumer Assessment of Healthcare Providers and Systems (HCAHPS) with a minimum of 968 observations per cell to accomplish this level of sensitivity. Our approach entails conservative oversampling (1500) to mitigate potential participant drop out. Inclusion criteria for the study is presented in Textbox 2.

### Study Activities

Below is a description of the study activities across 3 phases. See the Timeline section for a month-by-month depiction providing additional information about the study phases and timing of data collection activities.

#### Phase 1: Pre-Intervention

This phase of the study will begin immediately upon funding and is expected to take 9 months. In total, 20 patients and/or caregivers will be involved in the pre-intervention phase.

##### Obtain Institutional Review Board Approval

While the study is minimal risk, it does involve direct patient contact and will require institutional review board (IRB) approval. The IRB process at Ohio State University is conservative in its review of patient-contact research. As such, we propose to begin work on the IRB application upon notification of funding (pre-study) to allow sufficient time for securing approval.

##### Implementation Working Group Training and Activities

We have established an Implementation Working Group consisting of 3 patients, 4 providers (2 physicians and 2 nurses), and 2 content experts, as well as study investigators (see Innovation section). The Implementation Working Group will participate in a multi-week training program designed to encourage all members to voice their concerns equally and to orient group members to the study. Patient members of this group will receive US $150 in remuneration annually in recognition of the time involved with participation in this process. Physician and nurse participants will conduct this work as part of their obligation to engage in research as academic practitioners and thus will not receive additional remuneration for study participation.

The Implementation Working Group will develop the High Touch intervention materials building on the current training available in our pilot studies. Our intent is to establish a triage model in which patients receive materials matched to their comfort level in managing their care using MCB and MCA. We will gather user experience data to develop support materials for MCB through “Talk Alouds,” a common model for usability testing that helps to identify the elements of information necessary to support effective implementation [64]. In these sessions, users will work with tablet devices and be prompted to talk through their experiences with the technology. Participants’ utterances and the associated screenshots will be recorded, managed, and coded using Morae software [65]. The “Talk Aloud” process will collect participant information including demographics, as well as information about experience with technology, and technological resources.

The MCB platform will be examined using Nielsen’s heuristic principles [66] to identify aspects contrary to a user-friendly design. Usability testing will be conducted iteratively to optimize the intervention (see Timeline). We will first conduct individual sessions with 10 patient and caregiver volunteers with diverse levels of comfort with technology. These data will help to identify elements of MCB and MCA that will need particular attention in the High Touch intervention. The research team will then present the Implementation Working Group with a triage model for the High Touch and Low Touch intervention that can be aligned to individual patients’ levels of comfort and understanding, and support collaborative development of the intervention. Upon finalization of the Touch intervention material, we will conduct individual sessions with an additional 10 patient volunteers to pilot test the Touch interventions, gather feedback, and conduct a final revision of the materials accordingly.

##### Hire and Train Technology Navigators

We will utilize TNs to enroll patients in the RCT, administer admission and discharge surveys, and to deliver the High and Low Touch interventions. TNs will complete Collaborative Institutional Training Initiative (CITI) training and infection control training as needed for research that involves contact with patients. TNs will then be trained in the effective dissemination of the Phase 1 support materials and will serve as the primary conduit through which the High Touch interventions will be delivered to patients in participating inpatient units.

##### Identify Unit-Level Champions

For each of the 6 general medicine units participating in the study, we will identify a staff nurse champion to support the HT2 project. These champions will help promote the HT2 project.

#### Phase 2: Intervention

The intervention phase of the study is expected to be completed within 18 months. The targets for recruitment are 6000 patients and/or caregivers, and 100 providers.

##### Initial Contact: Patient and Caregiver Recruitment

Patients will be recruited based on the inclusion criteria presented in Textbox 2. Consent and completion of the admission survey (details below) will take place on a tablet device using Research Electronic Data Capture (REDCap), a secure Web-based application for building and managing online surveys and databases. At the conclusion of the survey, the tablet screen will display a visual cue (ie, the background color of the screen will change) indicating to the TN to which treatment group the patient has been assigned; this code will be indecipherable to the study participant. Based on the cue, the TN will initiate the appropriate study group intervention.

##### The High Tech/High Touch Intervention

Enrolled patients admitted to one of the 6 selected hospital units over the 18-month study period will be randomly assigned to one of the 4 study groups (see Figure 1).

##### Intervention at Discharge

Prior to the patient’s discharge, TNs will return to the patient room to activate the discharge survey on the tablet and collect the device from the High Tech groups. The TN will provide material necessary to create an MCA account if the patient does not have one or verify that the patient has access to their credentials before discharge (in case they forgot). As well, for those in the High Touch study groups, the TN will provide additional training in use of MCA as an outpatient. If a participant is re-admitted to the hospital, the TNs will again provide tablets to those patients in the High Tech group to enable continued study participation during the subsequent hospital stay.

##### Primary Data Collection

Both admission and discharge surveys will include questions on health literacy and experience with technology, and self-efficacy for managing chronic disease; the discharge survey will also include questions on patient satisfaction. In the discharge survey, a supplemental section for the High Tech groups assesses satisfaction with MCB and communication with staff in areas MCB may affect. When possible, question sets were used or adapted from existing sources. Table 1 lists the variables to be measured, the published source of the tool if applicable, the data collection instrument, and when the variable will be collected [67-69]. In addition, TNs will keep an ongoing weekly log of process measures, including variables such as the rate of patient refusal to participate, the number of participating patients who are discharged before the TN can return for the discharge survey, and problems encountered conducting the intervention.

**Table 1:** Variables to be measured.

Variable	Data collection instrument	Time of collection
Patient demographics and self-rated health status	Pre-survey and post-survey	At study assignment and at discharge
Chronic conditions and projected length of stay	EHR^a^	At study assignment
Self-efficacy to manage chronic conditions [67]	Pre-survey and post-survey	At study assignment and at discharge
Self-efficacy for using computers to access information (source: investigator)	Pre-survey and Post-survey	At study assignment and at discharge
Satisfaction with MCB^b^ (adapted from [68])	Post-survey	At discharge
Patient satisfaction [69]	Post-survey	At discharge
Rate of MCA^c^ use	EHR	6 months post-discharge
Information about the patients’ doctors (OSU^d^ versus non OSU for primary care and/or specialists)	EHR	6 months post-discharge
Readmission rate	EHR	6 months post-discharge

^a^EHR: electronic health record.

^b^MCB: MyChart Bedside.

^c^MCA: MyChart Ambulatory.

^d^OSU: Ohio State University.

##### Post-Intervention Data Collection

There are 4 types of post-intervention data collection: (1) 15-day post discharge patient interviews, (2) 6-month post discharge patient interviews, (3) interviews with providers and staff, and (4) and secondary data collection. All interviews will be audio recorded for transcription and to permit rigorous qualitative analysis. Patients (or caregivers) will be provided with gift cards as a token of appreciation for their participation in the interviews. In addition, we will contact patients who were discharged from the facility no less than 15 days post-discharge to request their participation in follow-up telephone interviews. Interviews will be conducted over the telephone and include a sufficiently large number of patients to ensure saturation of concepts across settings and experimental conditions. A semi-structured interview guide will be used including questions about the patient’s use of MCB or other technology while admitted and during the patient’s transition to the outpatient setting. We will also contact patients 6 months post discharge to request participation in phone interviews. These interviewees will be asked about their use of MCA or other patient portals since discharge, as well as asking about their experience with MCB and, if applicable, use of MCB during subsequent admissions. We will interview 20 providers (nurses and physicians) and all TNs every 4 months. Interviews will be focused on perceptions of changes in workflow, organizational culture, and patient interactions. Finally, secondary data collection will include abstraction from the EHR of participating patients and collection of de-identified metadata from university computer systems. This will provide information on frequency and type of use of both the MCB and MCA platforms. The Information Technology (IT) department, under the supervision of co-investigator Rizer, will provide these metadata. When patients are consented to join the study, the consent document will include a release of access to their EHRs for purposes of retrieving data at 6 months post-discharge on readmission rates, MCA use rates, and information on the patient's doctors (in health system versus out of network).

#### Phase 3: Analysis and Dissemination

##### Analytic Plan

We will employ a true mixed-methods research (MMR) model—one where both qualitative and quantitative data collection are employed to develop better theory. Creswell and Plano-Clark [70] define the central premise of MMR as “the use of quantitative and qualitative approaches in combination (that) provides a better understanding of research problems than either approach alone.” While we have identified aims reflecting the research questions guiding this project, we must also acknowledge that these questions have not been asked in this way. As a result, the process of piloting instruments and using accumulated knowledge to inform later components of the study is necessary in MMR. The qualitative effort is critical to achieving our specific aims because we need to understand the “why” behind what we are seeing in the data (ie, the context of variability).

##### Quantitative Data Analysis

As a first step, we will employ standard statistical tests (eg, analysis of variance, *t* tests) to compare patient and provider characteristics to examine whether the 4 cohorts are similar. While both multivariable regression and propensity score (PS) models are widely used to adjust for measured confounders and can be expected to yield similar findings, we plan to use a PS model.

We will construct a logistic regression model to predict use of MCA as a function of patient, provider, and covariate variables thought to be associated with the outcomes of interest. Across deciles of the PS distribution, we will compare factors associated with adoption (eg, age, comorbidities) between cohorts. To test for robustness, we will trim patients from the extremes of the PS distribution, refit the PS model, and evaluate overlap of the PS distributions (ie, common support) for the cohorts. Based on this evaluation, we will choose a strategy for matching, adjustment, or inverse weighting based on PS [71], and apply multilevel modeling (eg, hierarchical linear models). Co-investigator Huerta has published a number of studies using robust quantitative methods including significant work in the use non-parametric analytic approaches [72-75].

##### Qualitative Data Analysis

Qualitative analyses will be overseen by investigator McAlearney, a nationally recognized expert in qualitative methods, and will use the constant comparative method and a grounded theory approach to analysis [76]. Our iterative approach will involve reading interview transcripts, reviewing the literature, and discussing findings among investigators as the study progresses. This approach will enable us to explore emergent themes, and ensures saturation in data collection. Analysis will prioritize the elucidation of key concepts from individuals’ statements made in interviews (extraction), and conceptual development based on constant comparative analysis and the classification of data through code development [77,78]. The research team will use the ATLAS.ti software package [79] to facilitate coding and data analyses.

### Project Deliverables and Dissemination

Throughout the course of this project we intend to produce the following series of formal deliverables: (1) Interim Project Reports, summarizing preliminary findings at the end of each project year, (2) a Final Project Report summarizing and synthesizing findings across the project, and (3) a White Paper providing information about the HT2 interventions. We will also disseminate findings locally throughout OSUWMC, and broadly by producing a Public Webinar Presentation of Findings to be held at the conclusion of the project.

For further dissemination of research results, we will prepare and submit at least two peer-reviewed articles for publication in academic journals after concluding this research study, and we will seek to make presentations of findings at national meetings including Academy Health, and the Academy of Management, and the Healthcare Information and Management Systems Society (HIMSS). Further, we have included resources to support the submission of manuscripts to open source journals.

Beyond formal deliverables submitted to AHRQ, this project will also create products of value to a broad audience. Our Implementation Work Group training program will provide a structure that can serve as a model for any organization wishing to focus on these intervention areas, thus supporting the spread and long-term sustainability of this approach. In addition, our patient portal training materials (“High Touch”) will generate topic-specific materials that can be utilized by other organizations planning to implement patient portals and include patients in the process. Each of these products will be made available online upon completion of the project.

## Results

The proposed project has been funded as an Agency for Healthcare Research and Quality (AHRQ) R-01 study (#5R01HS024091-02). Data collection is underway. This research is expected to conclude in August 2019.

## Discussion

### Innovation

This study is groundbreaking. There are no randomized trials that have explored inpatient patient portals, in part because the technology itself has only recently emerged. However, we should expect these technologies will play a significant role in both inpatient and outpatient care in the future. While current patient portals have primarily been positioned to support the ambulatory care settings, as described earlier, the emergence of inpatient portals will open a new opportunity for engagement that may have implications across inpatient and outpatient settings.

#### Innovative Technology

OSUWMC is only the second health system in the nation to offer this highly innovative patient portal designed specifically for the inpatient environment. MCB provides patients with situation-specific information, such as daily schedule information, routing of questions to their care team, information about the care team, and immediate release of lab and test results. This will be the first large-scale study of an interactive tablet-based patient portal available at the inpatient bedside. This is a significant step toward using HIT to engage patients across the continuum of their care. OSUWMC has a strong relationship with Epic, and the research we propose will likely influence Epic implementations throughout the United States, thus potentially improving care delivery and outcomes nationwide.

Further, while health systems have not yet deployed such systems, the issue is more associated with an absence of tools provided by vendors as opposed to an unwillingness of health systems to implement. As the technology matures and more information comes online detailing the value proposition, these tools will see increasing use as well as greater diversity of tool availability (eg, tablet-based, mobile PHRs, etc). Further, as patients are better able to manage their disease states, they will “bend the cost curve” [80] by moving from high-cost interventions (eg, emergency room visits) to lower cost management of their conditions. The supposition is that the better we can engage patients in self-management of their own care and be effective at that practice, the lower the cost of care. Our study thus seeks to explore and gain a greater knowledge of how one such tool – an inpatient patient portal – can serve to not only influence outcomes related to the inpatient setting but throughout the entire continuum of care, including transitions and the outpatient care environment.

#### Compelling Experimental Design

While it would have been possible to conduct a smaller study that sought to explore the implications of patient portal adoption in greater detail, the need for larger-scale quantitative studies is without question. Existing studies have been resource limited and have not explored their implications in a RCT. As HIT tools such as patient portals become more prevalent, the likelihood of being able to execute a controlled trial is diminished. Rarely is a health system willing to subject the organization to such a broad scale intervention as Ohio State University is proposing.

#### Innovative Education Model

In addition, our Touch intervention will train patients not only in the technical aspects of utilizing the inpatient patient portal (MCB, the *how*) but also in the general use of an ambulatory patient portal (MCA) to manage their specific health condition over time (the *why*). Because the tablet MCB app allows an assessment of the patient’s comfort with technology and health literacy at the time of training, the Touch intervention can be tailored to the individual patient’s needs.

#### New Information Provided Through Subgroup Comparisons

Finally, our study will collect quantitative and qualitative data from a sufficiently large and diverse sample of patients to enable us to make comparisons across patient subgroups. We will assess the impact of our interventions in a variety of subgroups including those identified by demographics, by health condition, and by experience and comfort with technology. The results of this study will thus help health systems and providers understand what levels of technology and training work best to engage which patients.

### Limitations

Several limitations are inherent to this study, and we have explicitly tried to reduce the risks they pose. To address the possibility of missing data for the discharge survey, the TN will re-visit participating patients to activate the survey on the tablet and stay with patients during survey completion. This process should ensure that we have a high response rate. However, we do acknowledge that discharge can be unexpected and the TN will not be able to return to all participating patients before their discharge, particularly when discharge occurs sooner than planned or over a weekend. We attempt to mitigate this limitation with our recruitment protocol.

Currently, only two health systems in the United States are utilizing the Epic MCB product. While ambulatory patient portals are seeing a rise in use, inpatient portals have yet to emerge as a common engagement tool. This is due, in no small part, to requirements for meaningful use (MU) defined by the Office of the National Coordinator (ONC) as part of the Accountable Care Act. The stages that define MU have minimum participation requirements, and if a linkage between usage of MCB and MCA engagement is substantiated, we could see a rapid adoption of inpatient technology. Given that over 50% of US patients have their medical information stored within the Epic platform, the results of the present study may serve to either encourage or discourage (depending on the results) wider adoption and a proliferation of similar inpatient tools.

We have also taken steps to address platform and infrastructure changes. With such a long data collection period, there is a chance that the technology intervention may change as new functionality is brought online. Given the upgrade schedule at OSUWMC, we expect to see two updates to MCB as we await determination on the status of this proposal, and an additional update during the intervention. These pragmatic issues stand with any study, and the team is committed to addressing issues should they arise, in part by including leadership in the study, and by maintaining the Implementation Working Group throughout the data collection period.

### Strengths and Future Directions

The use of technology is an important ongoing issue in the study of how care is provided. With technology playing an ever-increasing role in the provision of care, tools such as patient portals offer another avenue through which behavior change can be facilitated. The proposed study will be the first RCT to examine the role that the new technology of inpatient portals could play to transform the way care is delivered.

Achievement of our HT2 study aims will lay the groundwork for future research and provide information to various health policy groups as well as increasing understanding about HIT implementation efforts for health systems. While qualitative studies offer insight into how and why things work, a determination of magnitude requires an experimental design such as an RCT. We expect this work will serve to provide information about how to integrate patient portals into practice, as well as about their contributions to care quality and their impact on readmissions, if such effects occur. Further, there are a number of hypotheses that could be tested within the context of this study outside of its primary focus that we are unable to enumerate due to space limitations. We expect these opportunities to be leveraged by doctoral students as part of dissertation work in the College of Public Health where Drs McAlearney and Huerta hold appointments and frequently serve on dissertation committees. Thus, this research can support practice innovation, researcher training, and future opportunities for training supplements.

## Abbreviations

AHRQ: Agency for Health Care Research and Quality
eHealth: electronic health
EHR: electronic health record
FAQ: frequently asked question
HIT: health information technology
HT2: High Tech/High Touch
IRB: institutional review board
MCA: MyChart Ambulatory
MCB: MyChart Bedside
MMR: mixed-methods research
MU: meaningful use
OSUWMC: Ohio State University Wexner Medical Center
PHR: personal health record
PS: propensity score
RCT: randomized controlled trial
TN: technology navigator
